# Jug r 6 is the allergenic vicilin present in walnut responsible for IgE cross-reactivities to other tree nuts and seeds

**DOI:** 10.1038/s41598-018-29656-4

**Published:** 2018-07-27

**Authors:** Pawel Dubiela, Stefan Kabasser, Nicolas Smargiasso, Sabine Geiselhart, Merima Bublin, Christine Hafner, Gabriel Mazzucchelli, Karin Hoffmann-Sommergruber

**Affiliations:** 1 Department of Pathophysiology and Allergy Research, Medical University of Vienna, Vienna, Austria; 2 Mass Spectrometry Laboratory, Molecular Systems Research Unit, University of Liège, Liège, Belgium; 3 Department of Dermatology, University Hospital St. Poelten, Karl Landsteiner University of Health Sciences, St. Poelten, Austria

## Abstract

Walnuts are ranked high in the list of the culprit foods inducing severe allergic reactions. Jug r 2 has been identified as a major allergen in common walnut by cDNA cloning from a somatic cell line. So far, studies were performed on the allergenic activity of recombinant Jug r 2, yet there is still no evidence about the physicochemical characteristics of the natural allergen. Therefore, we aimed to purify and deeply characterize natural Jug r 2 and to assess IgE cross-reactivity among vicilins from different tree nuts. Extensive mass spectrometry analysis of the obtained purified vicilin allowed identification of the protein sequence that displayed only 44% identity to Jug r 2. The newly identified vicilin (Jug r 6) was recognized by IgE of 26% in walnut allergic patients’ sera tested. In contrast to Jug r 2, Jug r 6 displayed a remarkable level of cross-reactivity when tested with homologues from hazelnut, sesame and pistachio. It is the first report showing the necessity of proteomic studies to improve allergy component resolved diagnosis.

## Introduction

Only a dozen of plant and animal derived foods account for around 90% of food allergic cases^1–3^. Among those, walnuts like other tree nuts are ranked high in the list of the culprit foods inducing severe reactions including anaphylaxis^4,5^. Allergies to milk and egg in infants are usually outgrown by the age of 3 years, but allergies to fish, shellfish, peanut, and tree nuts are usually permanent and often life-threatening, caused by a small number of allergens accounting for the majority of food hypersensitivity reactions in adults^6^. According to the EuroPrevall study walnut was ranked as one of the ten most frequently identified food allergen sources in Europe^3^. The frequency of walnut allergy in children with IgE-mediated food allergy has been reported as 4.2%^7^. Furthermore, allergic reactions to these nuts pose a serious health risk to affected individuals^8–10^ as shown by the UK anaphylaxis register, 5 out of 37 food-induced fatalities were caused by walnut^11,12^. To date, no registered immunotherapy for food allergy is available. Therefore, an accurate and reliable allergy diagnosis is essential to develop personalized dietary recommendations excluding the offending food(s) and still ensuring a balanced healthy diet.

Tree nuts are regarded a part of a healthy diet and are consumed as single food items, or as ingredients in a range of foods e.g. snacks but also increasingly added to dishes in varying concentrations. Tree nuts contain several allergenic seed storage proteins, 11S globulins, 7S globulins and 2S albumins in abundancy. These seed storage proteins share homologous amino acid sequences as well as 3D structural elements in botanically related plants^13^. For example walnut and pecan belong to the *Juglandaceae* family and cashew and pistachio are members of the *Anacardiaceae* family, respectively. Therefore, cross-reactivity among tree nuts is frequently observed^14^.

Nearly 2 decades ago the concept of component-resolved diagnosis (CRD) in allergy was first proposed^15^. This revolution turned allergen extract testing for the detection of serum derived specific IgE antibodies into diagnosis based on single allergenic molecule and raised many expectations for improved patients’ administration. CRD aimed the discrimination of cross-reactivity from genuine sensitization in allergic patients by using a multiplex testing^16^. The knowledge on the molecular allergy is continuously evolving. However, in the case of walnut allergy, *in vitro* testing still lacks specificity and sensitivity and remains one of the major challenge for allergologists^17^.

So far, six allergenic proteins were identified from walnut: Jug r 1 (2S albumin), Jug r 2 (vicilin), Jug r 3 (non-specific lipid transfer protein), Jug r 4 (11S globulin), Jug r 5 (Bet v 1-homologue), and Jug r 7 (profilin; www.allergen.org). However, only Jug r 3 was identified as the natural protein and the primary sequence of the others was predicted by cDNA cloning.

Jug r 2 has been identified by Teuber *et al*. as a major allergen in common walnut (*Juglans regia*) and studies were performed with recombinant (r)Jug r 2^18^. Pastorello *et al*. identified walnut vicilin as a minor allergen, since only 9 of 46 allergic patients presented IgE reactivity with recombinant vicilin precursor A. Interestingly, individuals sensitized to rJug r 2 were exclusively allergic to walnut and the majority of those vicilin-sensitized patients had several episodes of anaphylaxis and/or glottis oedema, even after the ingestion of minute quantities of walnut present as a hidden allergen^19^. This finding is in good agreement with the observations described by Teuber *et al*., whose patients had life threatening reactions^18^. In addition to this, Pastorello *et al*. also verified that sensitization to vicilin can occur via the gastrointestinal route^19^, thus confirming a possible classification as a class I food or true food allergen. True food allergens are proteins that sensitize prone individuals via the gastrointestinal tract and symptoms are not restricted to cross-reactivity with inhalant allergens. Although, studies were performed regarding the allergenic activity of rJug r 2, there is still no clear evidence about the molecular characteristics on the purified natural counterpart. It is known that for some allergies this could be the reason of insufficient specificity and sensitivity of the testing^20^. Furthermore, the cross-reactivity pattern among tree nuts remains unclear. Finally, the IgE reactivity patterns obtained from walnut, using the allergen specific approach lacks specificity. Therefore, we aimed to purify and characterize natural vicilin in depth with the application of various biochemical and immunological methods including high resolution mass spectrometry techniques. Moreover, we intended to study natural walnut allergens to unambiguously determine a cross-reactivity marker to improve tree nut allergy *in vitro* diagnosis.

## Results

### Jug r 6, is the abundant allergenic vicilin present in walnut kernels

**Figure 1:**
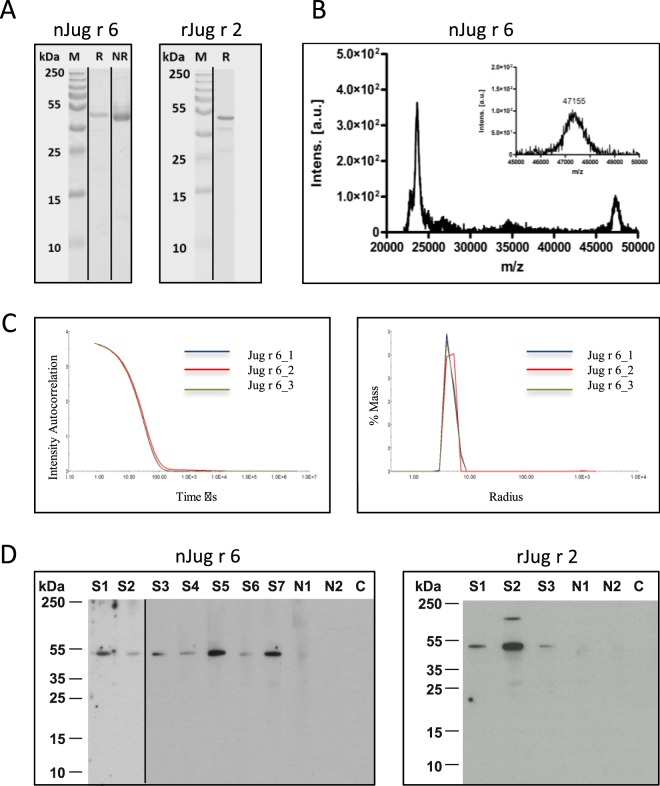
Characterization of nJug r 6 and rJug r 2. (**A**) The 15% Coomassie stained gels were run under the same experimental conditions and are shown as cropped gels; reducing (R), non-reducing (NR); (**B**) nJug r 6: MALDI-TOF and (**C**). Light scattering. (**D**) Immunoblot of purified nJug r 6 and rJug r 2. S1-S7 refers to patients’ serum samples. N1,2 and C represent normal human serua and secondary control, respectively.

**Figure 2:**
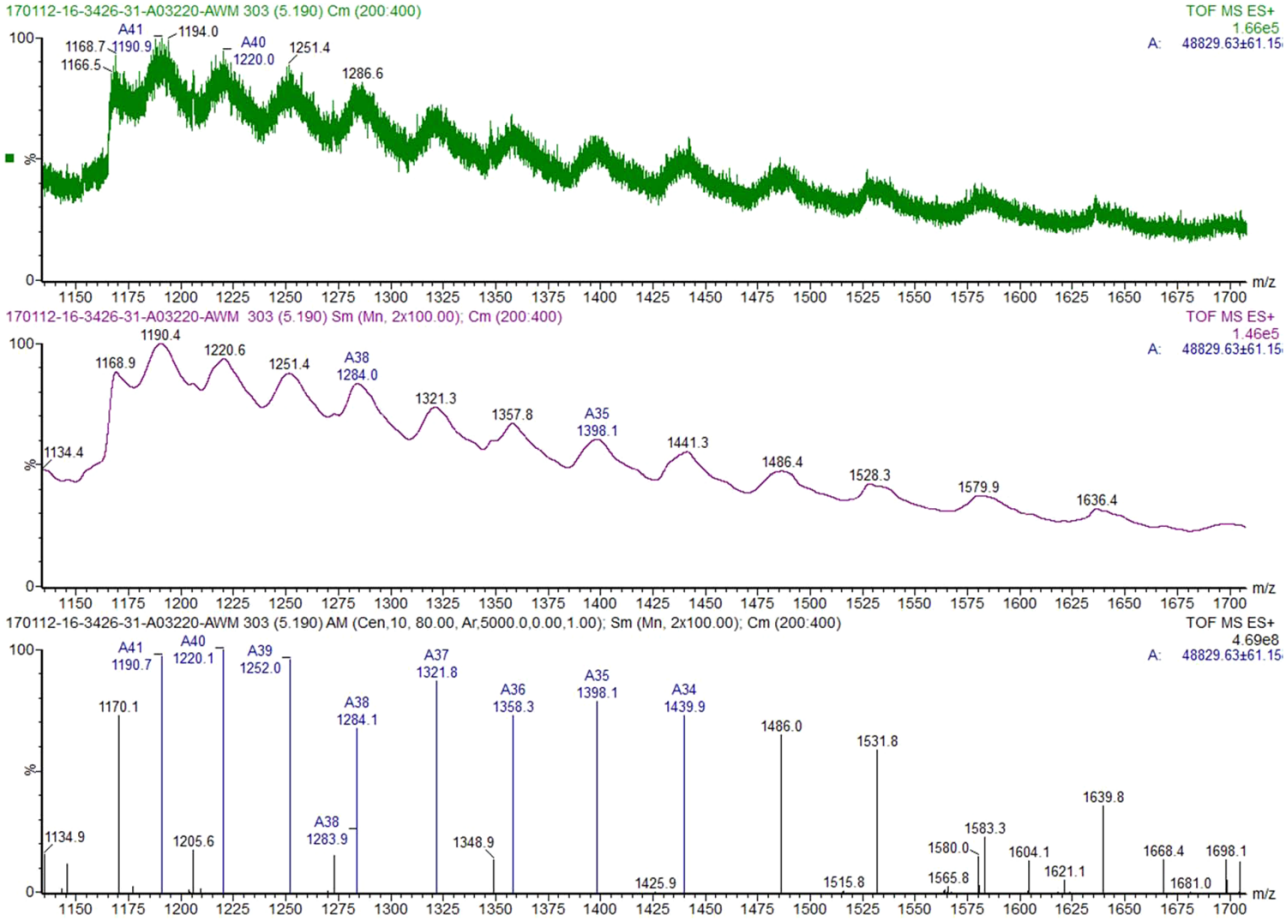
ESI-QTOF. Raw spectrum showing the protein multiply charged ions distribution (top spectrum). Smoothed (middle spectrum) and centered spectrum (bottom spectrum). The manual component finder algorithm (Masslynx 4.1) was used on the centered spectrum to determine the average entire protein mass 48,829 Da.

Natural vicilin was purified by using standard chromatography techniques, applying previous purification protocols established for vicilins. SDS-PAGE electrophoresis showed that the protein with a molecular mass of ∼50 kDa is highly pure and migrated as a single band (Fig. 1A). MALDI TOF-MS analysis of the protein (Fig. 1B) provided a mass of 47,155 Da and the ESI-QTOF experiments a mass around 48,829 Da (Fig. 2) corresponding to the predicted mass of 48,842 Da and 50,027 Da, for the non- and glycosylated protein, respectively (UniProt: PXD005744). Light scattering confirmed the high purity of vicilin that is present in the native state as a complex trimeric protein with a molecular mass of ∼135,658 Da calculated as an average of 3 measurements (Fig. 1C, Table E1). Natural vicilin displayed allergenic activity as shown by immunoblots using sera from walnut allergic patients. Sera were preincubated with HRP 100 (µg/ml) to rule out IgE recognition of glycan moieties (Fig. 1D).

**Figure 3:**
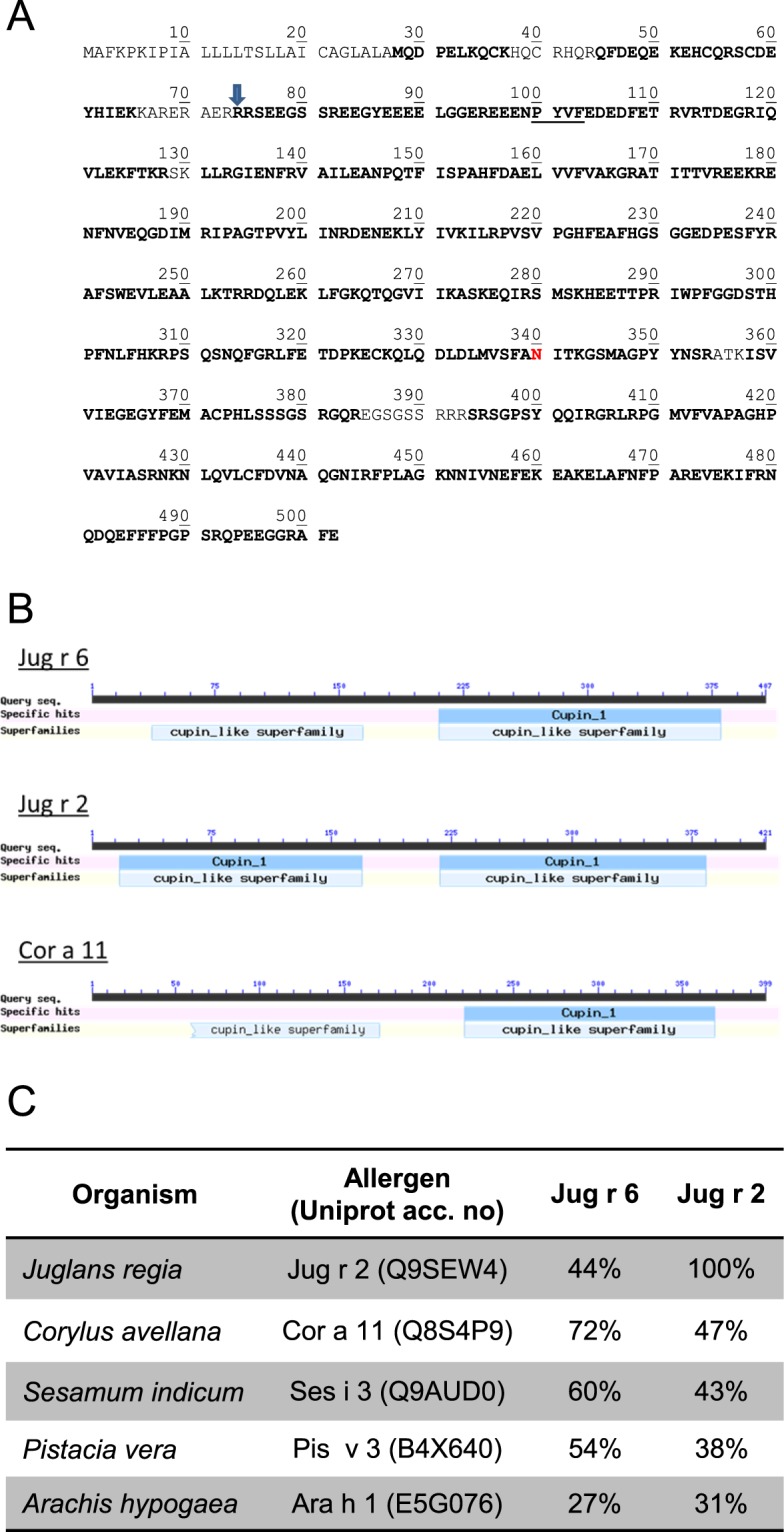
(**A**) Jug r 6 amino acid sequence (UniProt: PXD005744). The cleavage site for processing is indicated by an arrow. N340 in red corresponds to the glycosylation site. Residues in bold were confirmed by LC-MS/MS. Vicilin consensus sequence (cleavage site) is underlined; (**B**). Presentation of cupin domains; (**C**). Sequence identities shared by other allergenic vicilins.

A multi-enzymatic limited digestion (MELD) followed by LC-MS/MS analysis was used to sequence the peptides generated and identify proteins. We obtained a batch of purified vicilin which did not completely match with previously reported Jug r 2 but to another yet unknown vicilin. A full listing of the supporting peptides covering the identified vicilin sequence is provided in Fig. E1. Protein sequencing revealed 96.7% and 88.4% (peptide FDR < 1%, 94.6% and 87% (peptide FDR < 0.1%) or 93.9% and 82.6% (peptide FDR = 0%) of sequence coverage when considering both, the theoretical cleavage site in R73 (429 amino acids long) and the unprocessed form, respectively (502 amino acids long) (Figs 3A and E1). The protein described herein was submitted to the International Union of Immunological Societies’ (IUIS; www.allergen.org) Allergen Nomenclature Sub-Committee and upon positive evaluation was designated as Jug r 6^21^. In addition, analysis of another protein batch enriched in vicilins provided evidence of Jug r 2. However, the relative abundance of Jug r 2 was 7 times lower as compared to Jug r 6 (data not shown).

**Figure 4:**
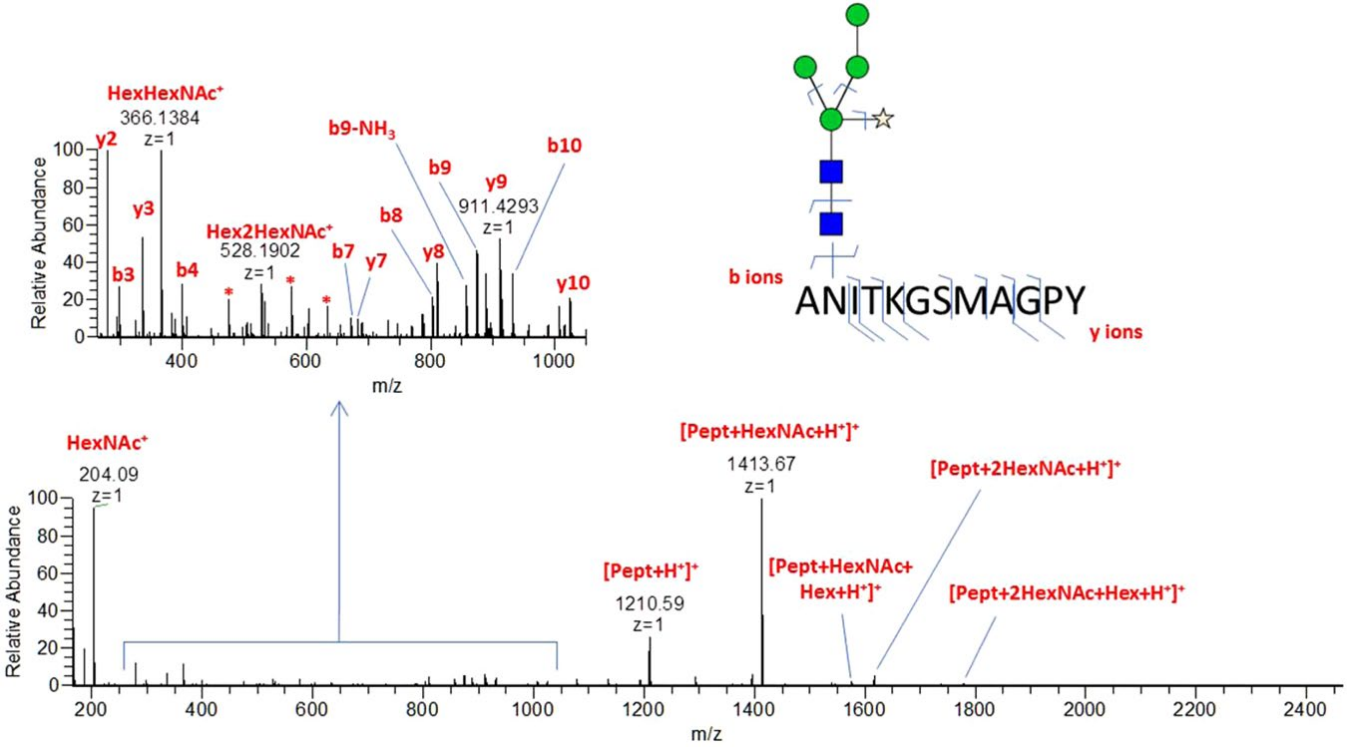
Annotated MS/MS spectrum of ANITKGSMAGPY-HexNAc2Hex4Xyl1 N-glycopeptide (precursor m/z = 1,198.504, z = 2). Picture insert proposes a potential glycan structure based on glycomod result. Oxonium ions (at m/z 204.09, 366.14 and 528.19) and fragments containing peptide + glycan fragments confirm the glycopeptidic nature of the ion. y and b ions are related to peptide fragments and confirm its sequence.

*In silico* analysis with NetNGlyc 1.0 Server predicted one N-glycosylation site at position 340 (data not shown). Occupation of this site was determined by semi-manual interpretation of the MS/MS spectra (Fig. 4).Two N-glycan compositions were deduced from LC-MS/MS data, corresponding to paucimannose structures displaying xylose. Based on MS1 glycopeptide signals, Hex4HexNAc2Xyl1 and Hex3HexNAc2Xyl1 relative abundances were found to be around 65% and 35%, respectively (Hexose 3/4 HexNAc 2 Pentose 1; Table E2). These results were further validated by the automatic search performed by PEAKS with 8 different peptide matches with a peptide FDR < 1%, and 6 when considering 0% FDR after having added the exact mass shift due to this specific glycosylated amino acid modification (Figs 3A and E1). Jug r 6 displayed cupin domains typical for the vicilin protein family (Fig. 3B). Comparison of amino acid sequence identity revealed higher similarity of Jug r 6 to Cor a 11 (72%), Ses i 3 (60%), Pis v 3 (54%) and Ana o 1 (51%) than to Jug r 2 (44%). Notably, Jug r 2 displayed also lower sequence identity to allergenic vicilins from tree nuts and seeds as compared to Jug r 6 but slightly higher to Ara h 1 (31 vs 27%; Figs 3C and E2).

All post-translational modifications detected in Jug r 6 (Fig. E1) have been deposited to the ProteomeXchange Consortium with the dataset identifier PXD005744 and 10.6019/PXD005744.

Recombinant Jug r 2 (rJug r 2) was produced in the heterologous *Pichia pastoris* expression system as a soluble protein with a yield of 5 mg/l. Ammonium sulphate precipitation and chromatography methods were applied for protein purification and delivered pure protein as shown by SDS-PAGE electrophoresis with molecular weight of ∼50 kDa (Fig. 1A). The intact N-terminal sequence of rJug r 2 was verified (REEEQQRH). IgE reactivity was tested by immunoblots using sera from walnut allergic patients (Fig. 1D).

### Jug r 6 displays intermediate thermal stability and susceptibility to enzymatic proteolysis

**Figure 5:**
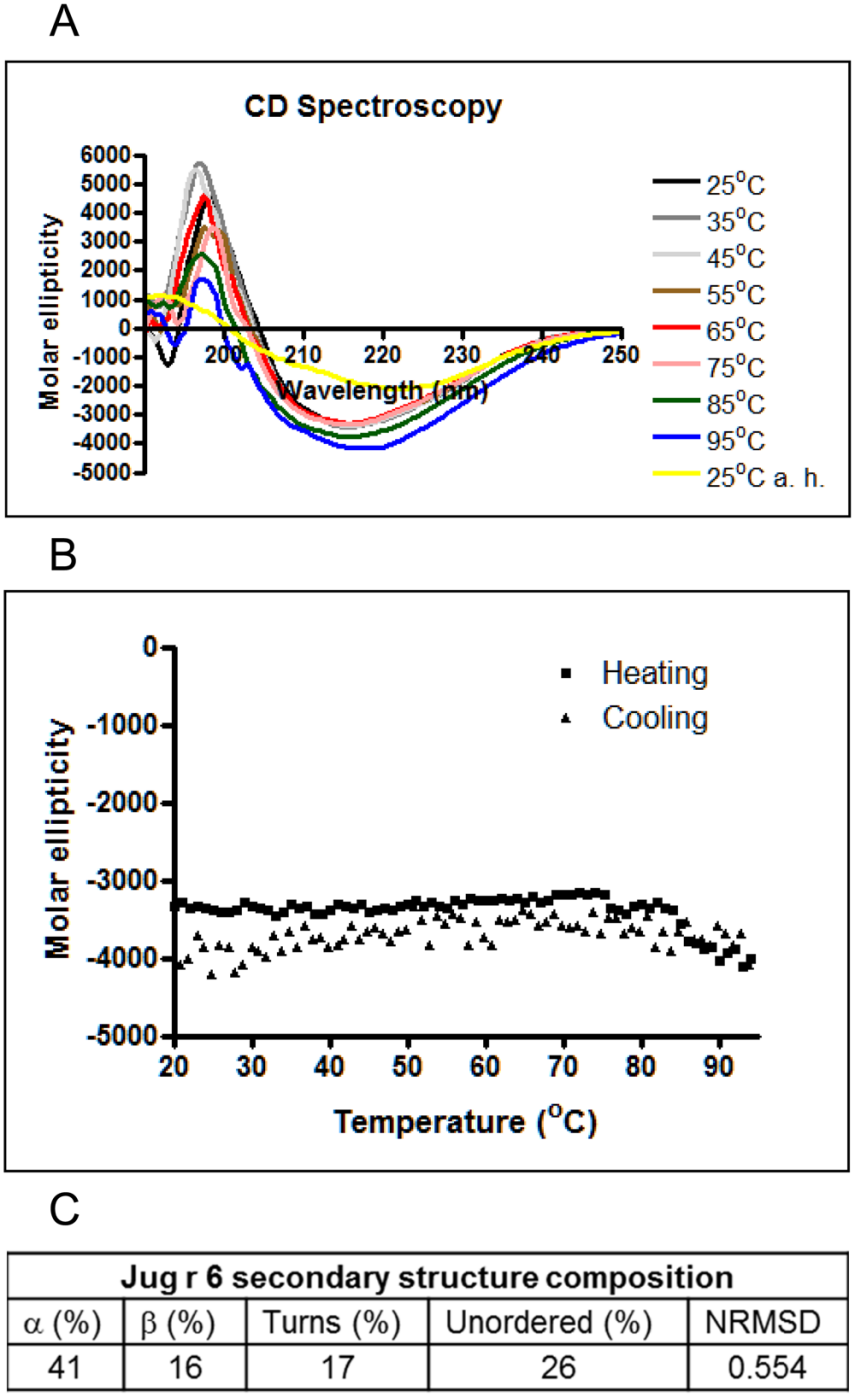
Far-UV CD spectra of nJug r 6: (**A**) Spectra of Jug r 6 at room temperature and each 10 °C till 95 °C, and subsequent cooling down to room temperature at pH 7.5; (**B**) Change in molar ellipticity at 218 nm during heating and cooling; (**C**) Secondary structure composition.

The overall secondary structure composition at room temperature and in relation to heat treatment was examined by CD spectroscopy (Fig. 5). CD spectra of the native protein showed the typical characteristic of β-sheet content with minimum and maximum at 218 nm and 196 nm, respectively. *In silico* calculations applying Dichroweb server^22^ revealed the composition of the secondary structure with 41% of α-helices and 16% of β-sheets (Fig. 3C). The intensity of the far-UV CD spectra decreased with temperature, and the shape of the spectra changed indicating that Jug r 6 unfolds and precipitates, which causes a decrease in the protein concentration in solution. Jug r 6 starts denaturation already at 45 °C and with increasing temperatures above 75 °C begins to precipitate, eventually becoming irreversibly denatured due to exposure of the hydrophobic core. When Jug r 6 is heated up to 95 °C and subsequently cooled down to 25 °C, the protein cannot be re-solubilized in aqueous solution (Fig. 5A, B).

**Figure 6:**
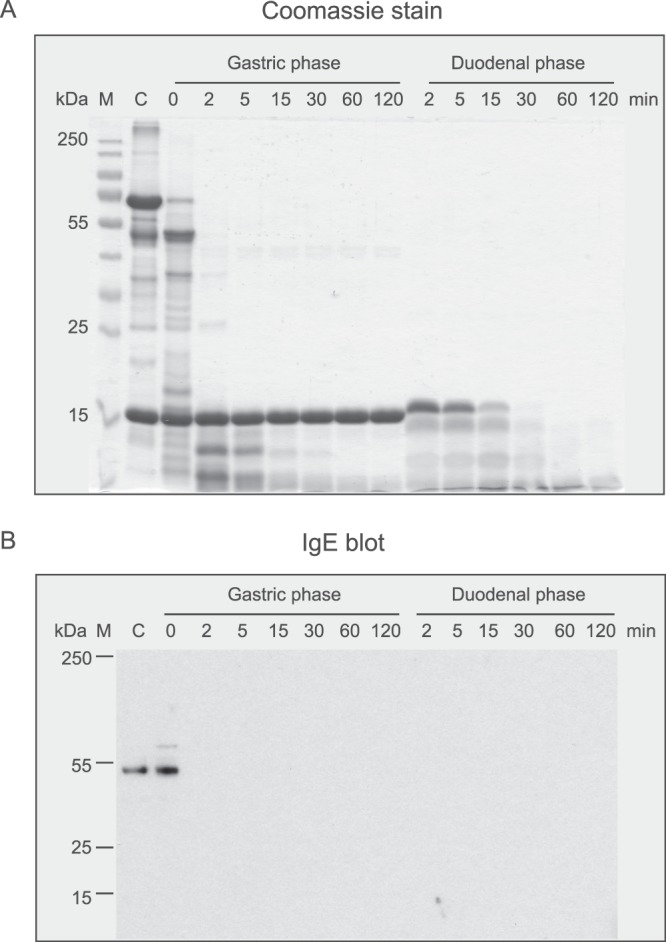
Effect of simulated gastric and duodenal digestion on purified nJug r 6. (**A**) Coomassie stained 15% SDS-PAGE of digested samples after the indicated times of gastric followed by duodenal digestion. BSA and Bos d 5 were used as controls for gastric and duodenal digestion, respectively; (**B**) IgE binding of digested samples using a pool of sera from walnut allergic patients.

The result of the simulated gastrointestinal digestion showed that Jug r 6 was susceptible to gastric digestion (Fig. 6). Already after 2 minutes of pepsinolysis we can observe full degradation of the protein. BSA was used as positive control of gastric phase and degraded in a similar way as Jug r 6 immediately after addition of pepsin. After two hours of gastric digestion the pH was raised to 6.5, and the intestinal enzymes trypsin and chymotrypsin were added. Bos d 5 was used as positive control for duodenal digestion. In line with earlier studies^23,24^, Bos d 5 was stable to gastric conditions but rapidly degraded during duodenal digestion (Fig. 6A).

### Jug r 6 contains allergenic activity

In the inhibition ELISA with HRP that blocked IgE specific for CCD, 20 of 77 patients (26%) demonstrated IgE binding to the purified Jug r 6 (data not shown).

**Figure 7:**
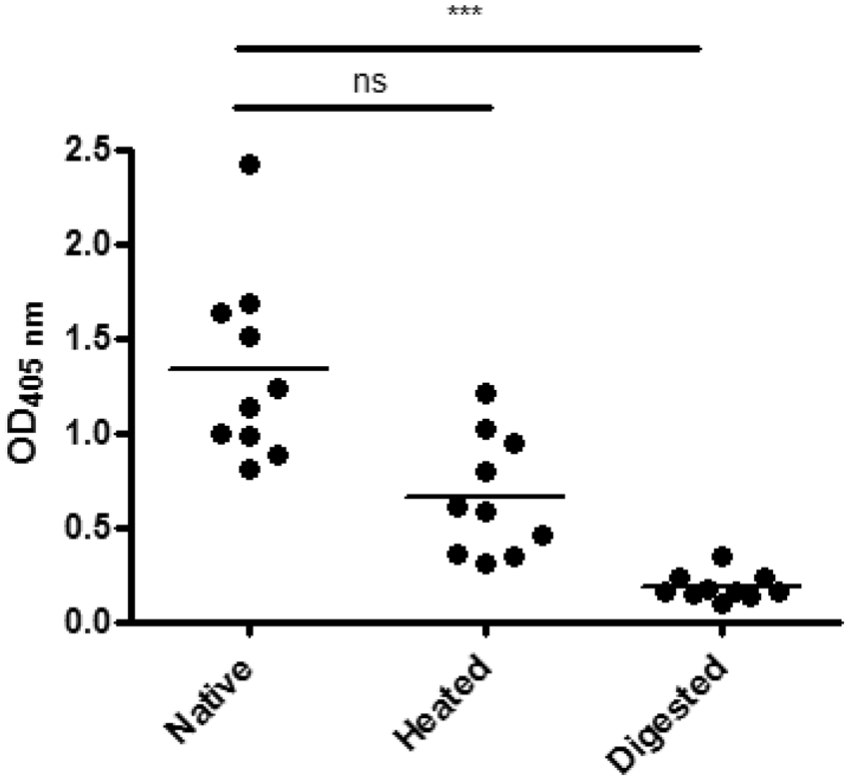
IgE binding of sera from walnut allergic patients to nJug r 6. Native, heated and digested nJug r 6 was tested for IgE-binding capacities. Dots represent the mean values for duplicates. The *p* value refers to the comparison between tested conditions. ***p ≤ 0.001. Not significant (ns) difference p > 0.05.

Ten pre-selected sera recognizing Jug r 6 were further tested for IgE binding to heat treated and gastrointestinally digested Jug r 6. The comparison of IgE recognition of native and digested sample revealed a significant difference calculated by one-way ANOVA Kruskal-Wallis Z Test (p-value ≤ 0.001, median 1.32 vs 0.18). Furthermore, IgE recognition of digested Jug r 6 was distinctly reduced if compared with native protein. Most of the signals were just above the threshold. This observation is supported by Western Blot analysis of samples obtained at indicated time points during digestion. IgE from patients’ serum pool recognized Jug r 6 only in the control sample and right after adding pepsin (Fig. 6B). However, digested Jug r 6 completely inhibited IgE binding to the intact protein (Fig. 7).

Heating of Jug r 6 also affected IgE binding as measured by ELISA. All sera displayed lower IgE binding capacity to the heated sample (Fig. 7). However, the difference did not reach statistical significance (p-value > 0.05, median 1.32 vs 0.66). Notably, the signal was approximately 3.5 fold higher than for the digested sample (median 0.66 vs 0.18).

### Jug r 6 is a marker of IgE cross-reactivity among tree nuts, and sesame

**Figure 8:**
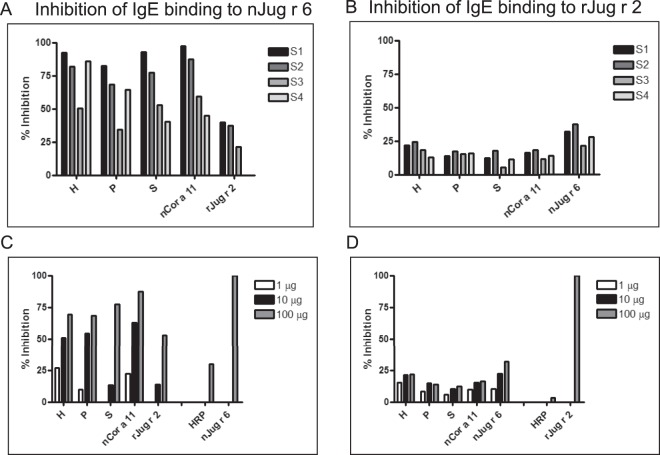
Inhibition IgE ELISA. (**A**,**B**) Inhibition of IgE binding to nJug r 6 and rJug r 2 by preincubation of walnut allergic patients sera (S1-S4) with 100 µg of rJug r 2, nCor a 11 and tree nuts and seed extracts, respectively; (**C**,**D**) Representative titration of tested inhibitors. H – Hazelnut; P – Pistachio; S - Sesame extracts. S1–S4 represent selected individual sera positive to Jug r 6.

Further, we investigated the level of cross-reactivity among nJug r 6 and related proteins from tree nuts and seeds (Fig. 8). In the experiments we included also recombinant rJug r 2, the previously reported vicilin from walnut. All 4 sera displayed reduced IgE binding to Jug r 6 when pre-incubated with nCor a 11 and extracts from hazelnut, sesame and pistachio, respectively. The highest level of cross-reactivity was detected for nCor a 11 and hazelnut extract followed by sesame and pistachio. Interestingly, preincubation with rJug r 2 revealed only limited inhibition for 3 sera whereas 1 serum remained unaffected. IgE recognition of related vicilins was patient-specific and varied between 59% and 97% for nCor a 11 and 51% and 92% for hazelnut extract. Similar results were obtained for sesame (45–93%) and pistachio (34–83%). Inhibition by rJug r 2 varied between 21% and 39% (Fig. 8A,C). Additionally, experiments with reaction between walnut patients’ allergic sera and Jug r 2 blocked by nJug r 6, Cor a 11 and tested extracts were performed and revealed much lower cross-reactivity than for nJug r 6. All of tested proteins and extracts slightly inhibited reaction with rJug r 2. The highest level of cross-reactivity was detected for nJug r 6 (21–37%) followed by hazelnut extract (12–25%) and nCor a 11 (11–18%). Blocking by pistachio and sesame remained low and varied between 14–18% and 5–17%, respectively (Fig. 8B,D). Purified nCor a 11 and extracts (hazelnut, sesame and pistachio) used for inhibition ELISA are shown in the Fig. E3.

## Discussion

In food allergy extract based *in vitro* diagnosis frequently lacks specificity and sensitivity. This can be overcome by the complementary application of allergen specific (component resolved) diagnosis. However, it is evident that some allergen tests lack important components. This is a known problem for walnut allergy where it seems that the allergen panel needs additional components, to facilitate the patient tailored management of their food allergy and to assess the risk of cross-reactions to other tree nuts and seeds. Vicilins are important, cross-reactive tree nut allergens identified in the majority of plant-derived foods and are often associated with severe symptoms in food allergy^25–27^. Jug r 2, the 7S vicilin-like protein, from English walnut, was described as a major allergen and its sequence was investigated by cDNA cloning from a somatic embryo cell line^18^. So far, Jug r 2 related studies were performed with the recombinant protein and data on the natural Jug r 2 were missing^18,19,28,29^. Furthermore, none of the walnut allergen was shown to be a reliable marker for cross-reactivity among tree nuts.

We aimed to purify and deeply characterize vicilin in walnut kernels on the protein level. Following purification, the obtained samples were sequenced with highly sensitive LC-MS/MS methods. Surprisingly, the peptides did not match the sequence of Jug r 2 but were identical to another protein present in the recently published/released walnut transcriptome database^30^. Our data clearly showed that this vicilin is present in walnut kernels and is recognized by IgE antibodies from allergic patients’ sera. This newly identified allergen was designated Jug r 6 by the IUIS allergen nomenclature committee. Jug r 6 is responsible for cross-reactivity with its counterparts from hazelnut, sesame and pistachio. Moreover, applying our extraction and purification methods we also detected natural Jug r 2 on the protein level in vicilin enriched batches, yet at considerably low concentration levels as compared to Jug r 6. In order to compare the allergenic activity of Jug r 2 and Jug r 6, respectively we expressed rJug r 2 in *P*. *pastoris* to compare it with the newly identified natural vicilin to assess potential cross reactivity.

Analysis performed with LC-MS/MS and *in silico* approaches provided sequence information. Jug r 6, as other members of vicilin family (Cor a 11, Ara h 1), possesses the cupin domain and a conserved glycosylation site with glycan composition: Hexose: 4 N-acetyl hexosamine: 2 Pentose: 1^27,28,31,32^. The sequence identity among homologues from related tree nuts (hazelnut, pistachio and cashew) and seeds (sesame) is even higher than for Jug r 2 that seems to be closer related to Ara h 1.

As other vicilins, Jug r 6 is represented in the native state as a complex trimeric protein^33^. Its secondary structure, similar to the homologue from hazelnut, Cor a 11^31^, is composed of a mixed population of α-helices and β-sheets and presents, typical for its protein family^34^, intermediate heat stability. Considering the resistance to the gastrointestinal digestion, Jug r 6, similarly to Ara h 1, was susceptible to immediate proteolysis^35^.

Further, we investigated the immunological properties of this new vicilin. Jug r 6 was recognized by specific IgE antibodies in 26% of the walnut allergic patients’ sera. It corresponds to the study on Cor a 11 from hazelnut, and Pis v 3 from pistachio that are considered as minor allergens that were recognized by 47% and 37% of patients, respectively^31,36^.

The presence of conformational and linear epitopes was studied by stability assays followed by IgE ELISAs. Based on our heating results, Jug r 6 contains both, linear and conformational epitopes.

**Figure 9:**
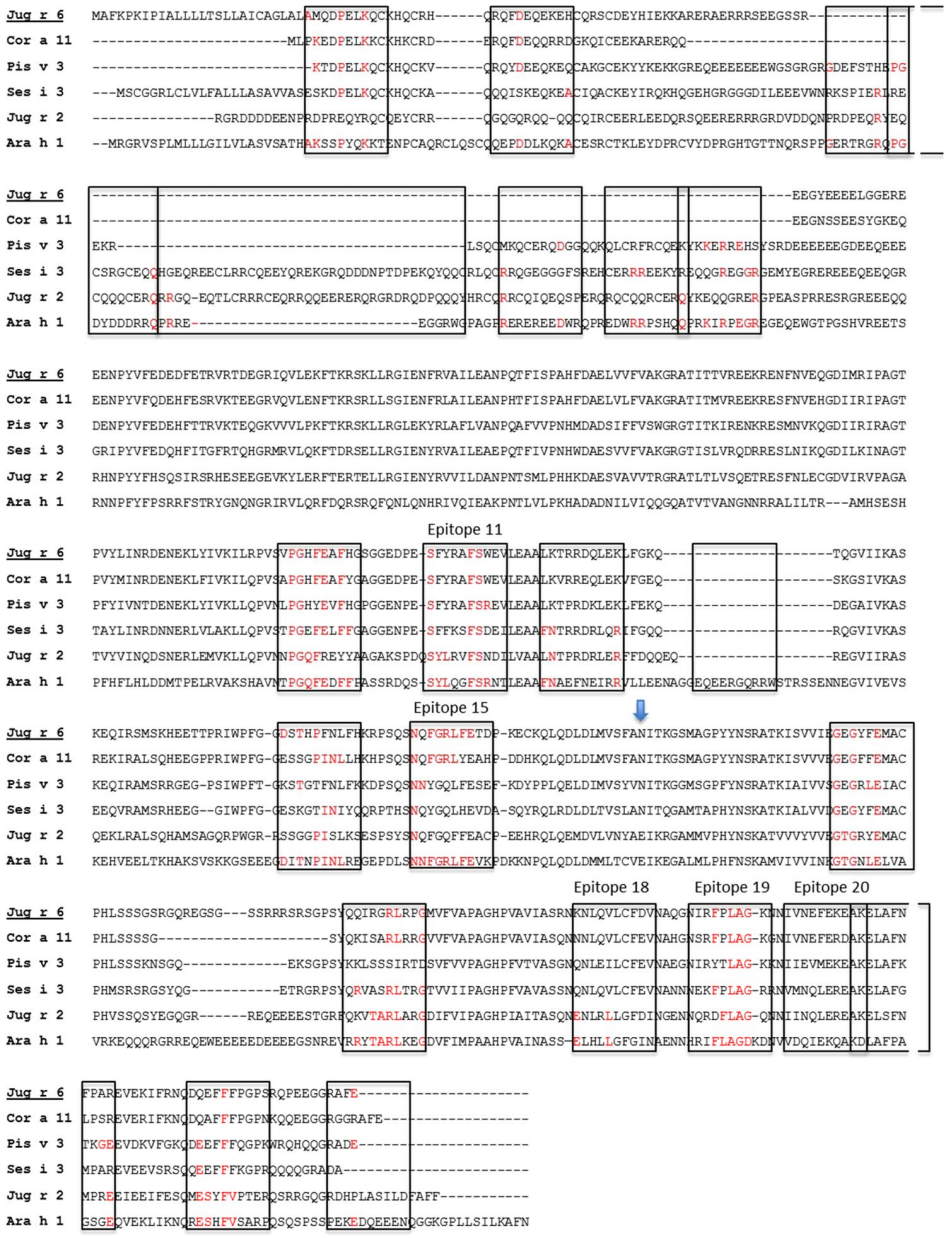
Sequence alignment of Jug r 6, Cor a 11, Pis v 3, Ses i 3, Jug r 2 and Ara h 1. The putative N-glycosylation site is indicated by vertical arrow. Linear B-cell epitopes mapped for Ara h 1^37^ are marked with the open boxes. Protein surface epitopes^25^ are marked with numbers. The alignment was performed with CLUSTAL-W 2.1.

Furthermore, we investigated the level of cross-reactivity between Jug r 6 and Jug r 2 and related allergens. As sequence identity suggested, the highest level of common epitopes was found for Cor a 11, followed by Ses i 3, Pis v 3 and Jug r 2. Sequence alignment between Jug r 6 and other vicilins from tree nuts (Cor a 11, Ses i 3, Pis v 3 and Jug r 2) and Ara h 1 revealed high sequence similarity and the presence of some well conserved residues in epitope regions known for Ara h 1^37^. Notably, some of the conserved epitopes represent residues present on the protein surface as mapped by Barre *et al*. (Fig. 9)^24,25^. Therefore, our data indicate that Jug r 6, opposite to Jug r 2, can be considered as a marker of cross-reactivity between walnut and hazelnut, sesame and pistachio.

In summary, we identified a new walnut allergen, Jug r 6. The allergen shares the biochemical features of the vicilin family. Our data provide evidence that Jug 6 is a closer homologue to Cor a 11, Ses i 3 and Pis v 3 than Jug r 2. Therefore, it can be considered as a marker for cross-reactivity among other tree nut vicilins in component resolved diagnosis. In this context, addition of Jug r 6 to the walnut allergen panel for component resolved diagnosis is recommended. The application of high resolution proteomic techniques enabled us to unambiguously identify the primary sequence of nJug r 6 and to assess post-translational modifications. These highly sensitive methods should be frequently used to characterize individual proteins and their modifications relevant for their allergenic activity. Since most of the allergen sequences were investigated in the 90s with the application of cDNA cloning, their primary structures and posttranslational modifications should be reconsidered to further improve sensitivity and specificity of the testing. It was shown for *Chlamydia* species antigens that the proteomic approach lead to identification of 75 new antigens (of 185 known) and many of previously described molecules were restructured^38^. As comprehensively revised by Shreffler *et al*. the current allergen characterization is probably significantly biased and our study is the first one looking for a new direction, namely including already established highly refined approaches such as mass spectrometry to overcome previous shortcomings^39^.

## Materials and Methods

### Chemicals and reagents

All reagents were purchased from Sigma-Aldrich (Saint Louis, MS, USA) unless stated otherwise.

### Purification of Jug r 6

Purification of walnut vicilin was designed based on previous protocols^40^. Kernels were heated (100 °C; 3 min.) and ground in a kitchen blender followed by defatting three times for one hour at room temperature using n-hexane in a ratio of 1:3 (w/v). Subsequently, proteins were extracted from dried flour (1:50 w/v) with 20 mM Tris buffer, pH 8.0, containing 0.5 M sodium chloride, protease inhibitors (Roche Diagnostics GmbH, Mannheim, Germany) and 5% (w/v) poly-(vinylpolypyrrolidone) for two hours at 4 °C. The vicilin was purified applying 3 consecutive chromatographical steps consisting of ConA affinity, anion exchange and gel filtration based separation. The ConA chromatography was performed with Concanavalin A Sepharose (self-packed column, 5 ml volume, Thermofisher, Little Chalfont, UK) and after loading, unbound proteins were collected, and separated from bound proteins (extraction buffer containing 0.4 M methyl α-D-mannopyranoside). The flowthrough was dialyzed against anion exchange running buffer (20 mM Tris, pH 8) and loaded onto a Mono Q 5/50 GL column (Thermofisher). Bound proteins were eluted with (0–0.5 M NaCl) linear gradient. Fractions containing proteins of interest were pooled, concentrated and further separated by gel filtration (BioSEP SEC-S2000 column, Phenomenex, Torrance, CA, USA). The column was equilibrated with 20 mM Tris pH 7.8 containing 0.25 M NaCl, pH 7.8 and 200 µl of the concentrated sample was loaded onto the column. Proteins were eluted with the running buffer over 1.5 column volumes and the obtained fractions were analyzed by 15% SDS-PAGE and western blotting.

### Jug r 2 production in *Pichia pastoris*

The DNA sequence of the mature Jug r 2 was retrieved from the proteins’ precursor cDNA (GenBank: AF066055.1) starting at nucleotide position number 514 and supplemented with the endoplasmatic reticulum - retention signal (KDEL) at the C-terminus. Codon optimization, gene synthesis and cloning with KpnI and EcoRI to the pPICZα vector were performed by Thermofisher Scientific GENEART GmbH (Regensburg, Germany). Plasmid was linearized with SacI and used to transformed electrocompetent GS115 *Pichia pastoris* cells. Multi-copy clones were screened by replica plating and inoculated to 200 ml of MGYH (minimal glycerol medium containing histidine) containing 25 µg/ml zeocin. After 24 h of incubation at 28 °C under constant shaking 220 RPM, culture reached OD_600_ of 2.0, cells were harvested and placed into 200 ml of MMH (minimal methanol medium + histidine). The expression were maintained for 5 days at 24 °C, 140 RPM with addition of 1% (v:v) methanol.

For protein isolation, 2 g of wet cells were resuspended in 10 ml of Yeast Protein Extraction Reagent (Thermofisher Scientific, Rockford, USA) and cell disruption was performed using the Microfluidicer LV1 (Microfluidics Corp., Westwood, MA, USA). To enhance lysis, the cell suspension was incubated for 20 minutes at 45 °C and 1350 rpm. Subsequently, cell debris were removed by centrifugation (8000 × g; at room temperature for 15 min.) and the lysate was dialyzed against 20 mM TRIS/HCl, pH 8.0. The solution was precipitated with 20% of ammonium sulphate (w:v) and after solubilization and re-dialysis as before applied to MonoQ 5/50 GL column (GE Healthcare Life Sciences, Little Chalfont, UK) followed by gel filtration BioSEP SEC-S2000 column (Phenomenex, Torrance, CA, USA) as described above for purification of the new walnut vicilin.

### Preparation of extracts from hazelnut, sesame and pistachio

Extracts containing vicilins from hazelnut, sesame and pistachio were prepared as previously described for purification of Cor a 11^40^. Briefly, unshelled raw nuts and seeds were purchased from a local supplier. Nuts and seeds were ground and nuts were defatted by stirring in five volumes w/v of hexane for 1 h at room temperature. Defatted flours (each 10 g) were extracted twice by stirring for 2 h at 20 °C with 50 mM Tris-HCl pH 7.0 containing 500 mM NaCl, 6.2 mM sodium azide and protease inhibitor (2 tablets/100 mL, Roche Complete Protease inhibitor tablet) (1:10 w/v).

### Purification of Cor a 11

Following extraction, nCor a 11 was purified as described above for purification of the new walnut vicilin.

### Physicochemical characterization

#### N-terminal EDMAN degradation

The N-terminal sequence of rJug r 2 was determined using an Applied Biosystems Procise 491 sequencer (Applied Biosystems, Foster City, CA, USA). Purified protein (100 pmol) was adsorbed onto a Prosorb cartridge and subjected to sequence analysis.

#### MALDI-TOF

For intact mass determination, 2 µg of non-reduced nJug r 6 was spotted in a ratio 1:1 (v:v) with matrix (α-cyano-4-hydroxycinnamic acid) onto a ground steel MALDI target plate and measured in linear mode on a MALDI-TOF mass spectrometer (Microflex, Bruker Daltonics, Bremen, Germany).

#### ESI-QTOF

The sample was ultrafiltrated using Amicon (Millipore) with membrane cut-off of 3 kDa, to remove salts and placed in ammonium acetate 25 mM as final solvent to obtain final protein concentration around 40 µM. The sample was analyzed by ESI-Q-ToF-MS in positive mode at a protein concentration of 12 µM, 30% ACN, 0.5% formic acid (final) in ammonium acetate 25 mM. Calibration was performed using clusters of phosphoric acid in m/z range 50 to 3500, corresponding to raw spectra m/z acquisition range. Spectra deconvolution was performed by MassLynx software 4.1 (Waters) using smoothing, centering and manual component detection algorithm. Even though the sample was desalted the generated spectrum is noisy with the presence of possible adduct species leading to a high accuracy error on deconvoluted spectrum.

#### CD spectroscopy

Circular dichroism (CD) spectra of purified nJug r 6 (0.2 mg/ml in 10 mM sodium phosphate pH 7.5 buffer) were measured from 190 to 250 nm on a Jasco J-810 spectropolarimeter (Jasco International Co., Hachioji, Tokyo, Japan) at 25 °C using a 1 mm path length quartz cell. The effect of heating (2 °C/min) was measured at 218 nm. Moreover, a full scan was performed each 10 °C (till 95 °C). Spectra represent the average of 5 accumulations collected at 100 nm/min with a 2 s time constant, 0.5 nm resolution, and sensitivity of ±100 mdeg. The secondary structure composition was calculated using the Dichroweb server (program: Selcon; reference set: SET 7 Optimized for 190–240 nm)^22^.

#### Dynamic light scattering

A solution of 1 mg/ml of nJug r 6 was filtered through a 0.22 µm filter. The three samples in the non-reduced state were analyzed using a DynaPro NanoStar dynamic light scattering instrument (Wyatt Technology, Santa Barbara, CA). Each trial consisted of 20 acquisitions lasting between 20 and 60 s. A regularization algorithm (Wyatt DYNAMICS software) was used to plot the percent intensity scattered versus the hydrodynamic radius for each trial. The instrument is sensitive to samples in the range between ~5 Å and ~5000 Å. Molecular mass estimates were obtained based on the hydrodynamic radius.

#### LC-MS/MS

Two times 5 µg of protein content were independently processed as followed. Samples were placed in a solution of 50 mM ammonium bicarbonate, 1% sodium dodecyl sulfate (SDS) and heated for 5 minutes at 95 °C. Proteins were reduced for 40 minutes at 56 °C in 10 mM DTT then alkylated in 20 mM iodoacetamide at room temperature for 30 minutes before being precipitated using the 2D-Clean-up kit (GE Healthcare) according the manufacturer instructions. Both pellets were dissolved in a buffer containing 25 mM ammonium bicarbonate and 5 mM calcium chloride and digested using a Multi Enzymatic Limited Digestion (MELD). The MELD consists in two parallel 2 h digestions both using an optimized protease mixture. The mixtures are composed of the same proteases (Trypsin, Chymotrypsin and GluC) but in different relative quantities. 0.75 μg of each digest were pooled and the 1.5 µg were then injected on an Acquity M-Class UPLC (Waters, Milford, MA, USA) connected to a Q Exactive Plus (Thermofisher, Bremen, Germany), in nano-electrospray positive ion mode. The samples were loaded on the trap column (Symmetry C18 5 μm, 180 μm × 20 mm, Waters) in 100% solvent A (water 0.1% formic acid) during 3 minutes and subsequently separated on the analytical column (HSS T3 C18 1.8 μm, 75 μm × 250 mm, Waters); flow rate 600 nL/min, solvent A (0.1% formic acid in water) and solvent B (0.1% formic acid in acetonitrile), linear gradient 0 min, 98% A; 5 min, 93% A; 135 min, 70% A; 150 min, 60% A, total run time was 180 min. The MS acquisition was conducted in data-dependent mode. The parameters for MS acquisition were: MS range from 400 to 1600 m/z, resolution of 70,000, AGC target of 3e6 or maximum injection time of 200 ms. The parameters for MS2 spectrum acquisition were: topN of 10, isolation window of 1.6 m/z, normalized collision energy (NCE) of 28, resolution of 17500, AGC target of 1e5 or maximum injection time of 200 ms.

A database search was performed using Peaks Studio 7 (BSI), on an unreviewed-*Pentapetalae* Uniprot database (taxonomyA1437201, release 2016–02–25) supplemented with the new walnut vicilin full sequence based on the transcriptome data^30^ and the Jug r 2 mature sequence. The search parameters were: Parent Mass Error Tolerance: 3.0 ppm, fragment mass error tolerance: 0.015 Da, enzyme: none. For PTMs identification, we first searched semi-manually in LC-MS/MS data for N-glycosylations. Briefly, MS/MS were searched for the presence of known oxonium reporter ions. The presence of specific Y1 ion (fragment corresponding to [peptide + HexNac+ H+]+) was then checked in order to confirm glycosylation sites. For the matching spectra, Glycomod was used to generate potential N-glycans structures from parent ions mass (http://web.expasy.org/glycomod/). The relative abundance of the glycopeptides was determined by integration of MS1 chromatograms using Skyline 3.0.1. Spider search for PTMs: 485 built-in modifications and HexNac2Man4Xyl1 (N) (custom modification, delta monoistotopic mass = 1186.41). The results were filtered considering a peptide false discovery (FDR) of 1%, 0.1% and 0%.

### Simulation of gastrointestinal digestion

*In vitro* gastric (phase I) and subsequent duodenal (phase II) digestion of nJug r 6 was performed as described by Moreno *et al*.^41^ Purified Jug r 6 as well as controls (BSA and Bos d 5) were dialyzed against simulated gastric fluid (SGF) 0.15 M NaCl, pH 2.5 and diluted to a final concentration of 0.5 mg/ml, respectively. Pepsin (0.32% in SGF, pH 2.5) was added at a physiological ratio of enzyme/substrate (1:20, w/w) and digestion was performed at 37 °C. Aliquots were taken at scheduled time points (0, 2, 5, 15, 30, 60, and 120 min) and the reaction was stopped by increasing the pH to 7.5. Following gastric digestion, *in vitro* duodenal digestion was prepared by adjusting the pH to 6.5 and adding a bile salt mixture containing equimolar quantities (7.4 mM) of taurocholic acid sodium salt and glycodeoxycholic acid, 9.2 mM CaCl_2_ and 25 mM Bis-Tris, of pH 6.5. Finally, trypsin and chymotrypsin were added at physiological ratios of enzyme/substrate 1:400 and 1:100 (w:w), respectively. The digestion was performed at 37 °C with shaking and aliquots were taken after 2, 5, 15, 30, 60, and 120 min. Subsequently, samples were analyzed by 15% SDS-PAGE and immunoblotting using a serum pool from 10 walnut allergic patients. To demonstrate the functionality of the assay, the digestion was performed for single proteins as well as in a mixed assay format. While the mixed assay was used for the SDS-PAGE analysis and the immunoblot, single Jug r 6 digestion was subsequently used for the IgE ELISA experiments.

### Patients

Serum samples were obtained from a well-defined group of 77 walnut-allergic patients (43 female and 34 male patients with a mean age of 27.4 years) and 4 controls from non-atopic donors. All of the patients (n = 77) were recruited according to previously experienced food allergic symptoms (angioedema, urticaria, rhinitis) after walnut consumption and classified by allergologists as clinically allergic to walnut. Standardized interview was conducted to assess allergic symptoms to walnut. Subjects were also asked whether they could conclusively attribute their symptoms to walnut or whether they ingested a meal with multiple tree nuts. Patients were tested by ImmunoCAP and/or Skin Prick Test with positive results to walnut extract. They were type 1 allergic to walnut. The study was approved by the Ethics Committee of Lower Austria (GS4-EK-4/242–2013) and conducted in accordance with the Declaration of Helsinki. Patients gave written informed consent. All experiments were performed in accordance with the relevant guidelines and regulations. More detailed information about 10 patients sera included for protein characterization is shown in Table 1.

**Table 1:** Clinical data of 10 selected walnut allergic patients.

Patient no	Sex	Age	IgE specific for walnut (kU/L)	AE	URT	RHIN
1	F	19	4.8	+	+	
2	F	21	7.5	+		+
3	F	25	13.5	+	+	
4	F	28	26.0		+	
5	F	48	2.9	+	+	
6	F	23	6.4		+	
7	M	29	4.3		+	
8	M	21	3.8			+
9	M	24	9.2	+	+	
10	M	36	1.6		+	

AE, angioedema; URT, urticaria; RHIN, rhinitis.

### rJug r 2 and nJug r 6: immunological characterization

#### ELISA

IgE ELISA test was performed with native, heated (95 °C for 10 min) and digested Jug r 6. The purified samples were diluted with 50 mM Na-carbonate buffer, pH 9.6. A 96 well microtitre plate (MaxiSorb Immunoplate, Roskilde, Denmark) was coated with 100 µl of 2 µg/ml of the protein. The plate was incubated over night at 4 °C with 77 individual patients sera diluted in TBST + 1% BSA in different concentrations (1:10, 1:20 and 1:50) with 100 µg of HRP. Bound IgE was detected by incubation with 1:1000 diluted alkaline phosphatase-conjugated mouse anti-human IgE antibody (BD BioSciences, Heidelberg, Germany) for 2 h at room temperature, and color development was performed by using disodium p-nitrophenyl phosphate substrate tablets. OD was measured at 405 nm, and the mean value of the negative controls was subtracted. The One way ANOVA Kruskal-Wallis Z Test (Dunn’s Test) was used for comparison of IgE binding to native with heated and digested Jug r 6, respectively. P-values below 0.05 were considered statistically significant. Analyses were performed with GraphPad Prism software (GraphPad Software, La Jolla, CA, USA). All sera were tested in duplicates.

#### Western Blot

After separation by electrophoresis using a 15% polyacrylamide gel, proteins was blotted to a 0.45 µm nitrocellulose membrane (GE Healthcare, Uppsala, Sweden). Blotting was performed at 150 mA for 45 min. at 4 °C. Patients sera were diluted in TBS-T containing 1% BSA and 100 µg HRP as for ELISA. The secondary antibody, HRP conjugated goat anti-human IgE (KPL, Gaithersburg, MD, USA) was diluted 1:10000 in TBS-T + 1% BSA and incubated for 2 h at room temperature on the membrane. Blots were developed using the Clarity ECL Western Blot Substrate (Bio-Rad Laboratories, Inc.) following the manufacturers instruction. The membrane was placed in a light protected cassette (Hypercassette, Amersham, GE Healthcare, Japan) and exposed to an X-ray film (Amersham Hyperfilm™ MP; GE Healthcare, Japan) in the dark. Depending on the signal, films were exposed between 0.5 and 10 min and developed using a CP1000 AGFA film processor (Superior Radiographics, LTD., Madison, WI, USA).

#### Inhibition ELISA

The ELISA inhibition assay was used to test the ability of purified vicilins (rJug r 2, nCor a 11) as well as hazelnut, pistachio and sesame extracts to block the binding of specific IgE from walnut allergic patients’ sera to natural Jug r 6. Furthermore, the inverse inhibition with pre-incubation of Jug r 6 and detection of IgE against Jug r 2 was applied. The protocol was as for standard ELISA described above with additional sera pre-incubation (3 hours at RT) with tested inhibitors in concentration 1, 10 and 100 µg/ml. The inhibition of IgE was calculated as follows: (1-(OD_inhibited_-OD_control_)/(OD_uninhibited_-OD_control_)) × 100[%]. To validate the system, self-inhibition with 100 µg/ml Jug r 6 or Jug r 2 was performed.

### Data availability

The datasets generated during the current study are available from the corresponding author upon request.

## Electronic supplementary material

Supplementary information
