# Supplementary material for: Jug r 6 is the allergenic vicilin present in walnut responsible for IgE cross-reactivities to other tree nuts and seeds
Source: Sci Rep. 2018 Jul 27;8:11366. doi: 10.1038/s41598-018-29656-4 (PMC6063931; doi:10.1038/s41598-018-29656-4)
Supplement: Supplementary file 1 — Supplementary information [file 41598_2018_29656_MOESM1_ESM.pdf]

Jug r 6 is the allergenic vicilin present in walnut responsible for IgE cross-reactivities to other tree nuts and seeds

Pawel Dubiela, MSc[1], Stefan Kabasser, BSc[1], Nicolas Smargiasso, PhD[2], Sabine Geiselhart, PhD[1], Merima Bublin, PhD[1], Christine Hafner, MD[3], Gabriel Mazzucchelli, PhD[2], Karin Hoffmann-Sommergruber, PhD[1*]

[1] Department of Pathophysiology and Allergy Research, Medical University of Vienna, Vienna, Austria

[2] Mass Spectrometry Laboratory, Molecular Systems Research Unit, University of Liège, Liège, Belgium

[3] Department of Dermatology, University Hospital St. Poelten, Karl Landsteiner University of Health Sciences, St. Poelten, Austria

*Corresponding author: Karin Hoffmann-Sommergruber, PhD

Email: karin.hoffmann@muv.ac.at

Department of Pathophysiology and Allergy Research

Medical University of Vienna

Waehringer Guertel 18-20

A-1090 Vienna, Austria

Figures:

Fig. E1

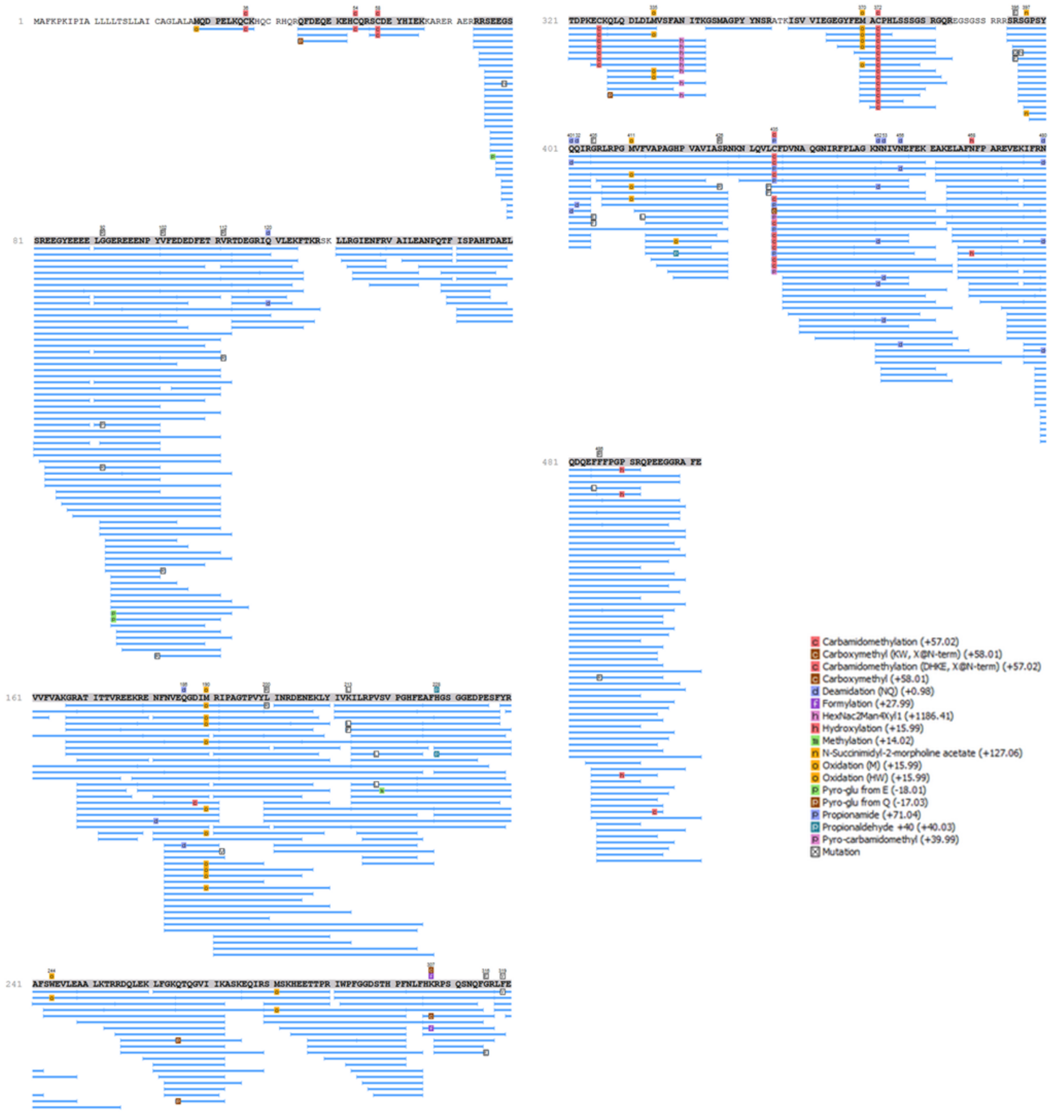

Figure E1 Protein identification of Jug r 6. Jug r 6 protein coverage view from the results filtered with a peptide FDR<1%. The protein coverage view maps the 481/455 peptides/unique peptides identifications onto the protein sequence. Each blue bar indicates an identified unique peptide sequence. Peptide identifications with the same amino acid sequence and the same PTMs are grouped together and displayed as a single bar. PTMs and mutations are highlighted with colored icons and white letter boxes. Highly confident PTMs and mutations are displayed on top of the protein sequence.

Fig. E2

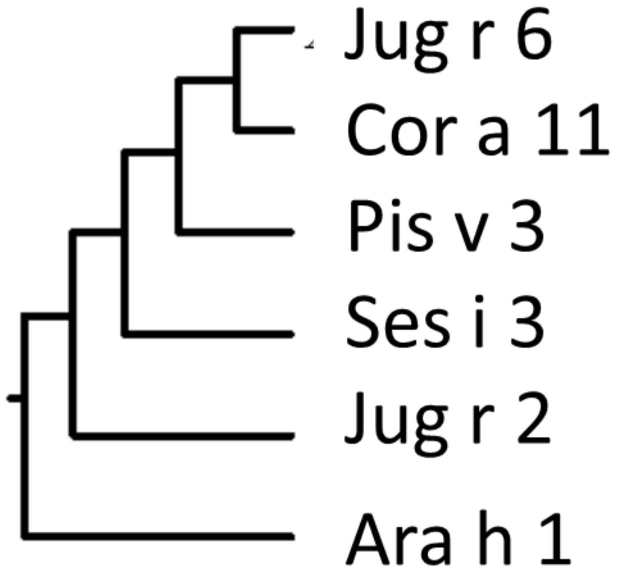

Figure E2 Protein phylogenetic tree based on sequence alignment. The alignment was performed with CLUSTAL-W 2.1

Fig. E3

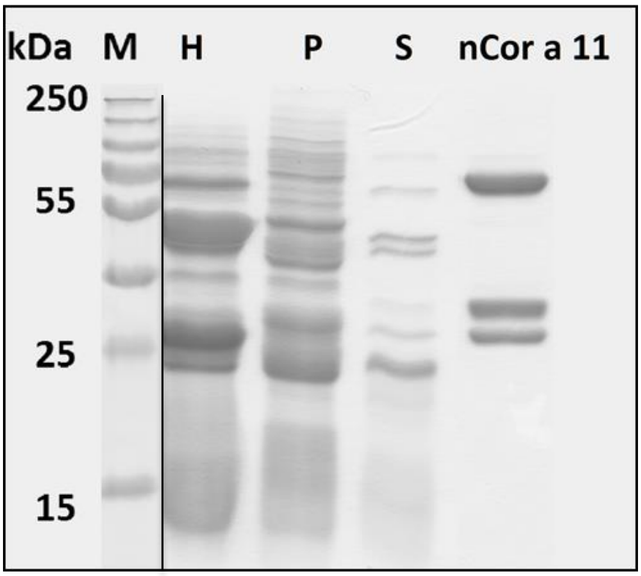

Figure E3 Proteins and extracts used for IgE ELISA inhibitions: H (hazelnut), P (pistachio), and S (sesame) extracts.

Table E1 Summary of data obtained with light scattering for nJug r 6.

ID	Sample	Acq Time (s)	Mw-R* Model	Radius (nm)	Diameter (nm)	Mw-R (kDa)
**1**	Jug r 6	5	Globular Prot.	4.73411	9.46822	127.968
**2**	Jug r 6	5	Globular Prot.	5.0313	10.0626	147.56
**3**	Jug r 6	5	Globular Prot.	4.78867	9.57735	131.446

*Mw-R – Molecular weight-Radius

Table E2 N-glycans relative abundances found on N340, based on MS1 signals.

Peptide	Glycan	%
ANITKGSMAGPY	HexNac2Hex3Xyl1	37
ANITKGSMAGPY	HexNac2Hex4Xyl1	63
ANITK	HexNac2Hex3Xyl1	34
ANITK	HexNac2Hex4Xyl1	66

Due to multienzymatic digestion, two peptides (ANITK and ANITKGSMAGPY) carrying N-glycans were observed. Similar proportions were found for both.
